# The Use of Fish Oil with Warfarin Does Not Significantly Affect either the International Normalised Ratio or Incidence of Adverse Events in Patients with Atrial Fibrillation and Deep Vein Thrombosis: A Retrospective Study

**DOI:** 10.3390/nu8090578

**Published:** 2016-09-20

**Authors:** Rebecca Pryce, Nijole Bernaitis, Andrew K. Davey, Tony Badrick, Shailendra Anoopkumar-Dukie

**Affiliations:** 1 School of Pharmacy Griffith University, Gold Coast, Queensland 4222, Australia; rebecca.pryce@griffithuni.edu.au (R.P.); n.bernaitis@griffith.edu.au (N.B.); a.davey@griffith.edu.au (A.K.D.); 2 Menzies Health Institute Queenslands, Griffith University, Gold Coast, Queensland 4222, Australia; 3 Royal College of Pathologists of Australasia Quality Assurance Programs, St Leonards, New South Wales 2065, Australia; tony.badrick@rcpaqap.com.au

**Keywords:** warfarin, fish oil, krill oil, bleeding, time in therapeutic range, atrial fibrillation, deep vein thrombosis

## Abstract

Background: Warfarin is a leading anticoagulant in the management of atrial fibrillation (AF) and deep vein thrombosis (DVT). Drug interactions influence the safety of warfarin use and while extensive literature exists regarding the effect on warfarin control and bleeding incidence with many medicines, there is little evidence on the influence of complementary medicines. The aim of this study was to assess the influence of fish and krill oil supplementation on warfarin control and bleeding incidence in AF and DVT patients. Methods: A retrospective analysis was conducted utilising patient information from a large private pathology clinic. AF and DVT patients receiving long-term warfarin therapy (>30 days) at the clinic and taking fish and krill oil supplements were eligible for study inclusion. Results: Of the 2081 patients assessed, a total of 573 warfarin users met the inclusion criteria with 145 patients in the fish and krill oil group (supplement group) and 428 patients in the control group. Overall, it was found that fish and krill oils did not significantly alter warfarin time in therapeutic range (TTR) or bleeding incidence, even when compared by gender. Conclusion: Omega-3 supplementation with fish and krill oil does not significantly affect long-term warfarin control and bleeding and thromboembolic events when consumed concurrently in patients managed at an anticoagulation clinic.

## 1. Introduction

Warfarin is an anticoagulant commonly used in indications such as the prevention of stroke in patients with atrial fibrillation (AF) and the treatment or prevention of deep vein thrombosis (DVT) [1]. Warfarin exerts its anticoagulant effects by interfering with the cyclical conversion of vitamin K and vitamin K epoxide. The action of warfarin is monitored utilising a method known as the International Normalised Ratio (INR) with a target therapeutic range of 2.0 to 3.0 appropriate for prophylaxis or treatment of venous thromboembolism (VTE) and reduction of the risk of systemic embolism in patients with AF [2,3]. A recommended measure of warfarin control is the time in therapeutic range (TTR) commonly measured by the Rosendaal linear interpolation method [4]. The clinical benefit and risks of warfarin are closely linked with a patient’s TTR with increases in TTR improving the risk-benefit profile of warfarin and leading to better patient outcomes [4,5]. According to a study by White et al. [6], patients with a poor TTR of <60% have a higher annual mortality rate, major bleeding rate, and stroke and systemic embolism rates than patients with good control (TTR > 70%). Guidelines vary in their recommended target TTR, with a minimum of 70% in European guidelines [7] but a minimum of only 60% recommended by local guidelines in Queensland Australia [8].

There are many factors that have been shown to influence warfarin control. Patient-specific factors, such as being female and being aged less than 50 years have been proposed to cause a decrease in TTR and warfarin control [9]. Furthermore, non-patient-specific factors causing changes to INR levels include major changes in diet or alcohol intake, systemic or concurrent illness, non-adherence to dosage regime, or drug interactions [10]. The incidence of drug interactions with warfarin is very high, greatly influencing the control and thus the safety of warfarin use. Although warfarin’s interactions with many medicines are well documented [11], information relating to complementary medicines is limited. Only two complementary or natural medicines (St John’ Wort and vitamin K) are listed in the Australian Medicines Handbook (AMH) [11] as having a known interaction with warfarin; however, a larger proportion of complementary medicines have limited or inconsistent evidence surrounding their potential to interact with warfarin therapy [12]. With a shift towards holistic approaches to healthcare, complementary medicine use has increased, with reports of two out of three Australians using complementary medicines [13]. Due to the proposed benefit of some complementary medicines in aiding cardiovascular disease [14,15], there is the realistic potential for warfarin patients, such as those with AF and DVT, to consume complementary medicines concurrently with warfarin. A retrospective analysis conducted by Ramsay et al. [16] assessed the pharmaceutical care plans for patients starting warfarin who attended an anticoagulation clinic in 2003. This study showed that 26.9% of patients were taking some form of complementary medicine, and approximately 60% of those patients were also identified to be taking complementary medicine that had the potential to interact with warfarin. While there is little complementary medicine that has documented interactions with warfarin, the vast majority has very inconclusive or conflicting data available.

Fish and krill oil products are among the many complementary medicines which has been flagged as having a potential interaction with warfarin [12]. Fish oil is the oil extracted from the tissues of oily fish, such as salmon, mackerel, sardines, and tuna [17], and krill oil is the oil extracted from the Antarctic krill *Euphausia superba* [18]. Contained within these extracted oils are two of three known long-chain omega-3 fatty acids—eicosapentaenoic acid (EPA) and docosahexaenoic acid (DHA) [19]. Due to the proposed mechanism of EPA and DHA, these products have been marketed to have benefits in aiding many conditions, including inflammatory disease (e.g., rheumatoid arthritis) and cardiovascular disease (e.g., the reduction of coronary heart disease) [19]. The physiological benefit of omega-3 fatty acids arises from their influence on prostaglandins, platelets, lipids, and thrombi [19,20,21] resulting in anti-inflammatory actions [22] and multiple cardiovascular benefits [23] including a reduction of blood pressure [24]. Australian guidelines support the consumption of omega-3 for protection from heart disease and stroke, with a recent study revealing no additional benefit nor adverse effects from further supplementation over the recommended 1 g per day [25]. Supplementation with fish and krill oil is widespread, with studies showing that these supplements are among the most widely consumed complementary medicines available [26,27]. It is due to this promoted reduction in cardiovascular disease that warfarin patients, such as those with AF and DVT, may choose to consume fish and krill oil supplements. Ramsay et al. [16] has already shown that warfarin and fish and krill oil supplements are being used together, with approximately 10% of their total study cohort consuming these products concurrently with warfarin.

Although several studies have been conducted assessing the change in incidence of bleeding events associated with fish and krill oil use [28,29], the effect of these supplements on warfarin control as measured by TTR is yet to be accurately determined. To date, very few studies have examined or reported on this effect. A review of recent literature (2000–2015) to ascertain this interaction only uncovered three clinical case reports [30,31,32]. In addition to being of the lowest level of evidence [32], the results obtained from these clinical reports were very conflicting. Two reports discussed an increase in INR following the initiation or increased dose of omega-3 supplementation [30,32], while the third discussed the occurrence of a major bleeding event without a relative increase in patient INR [31]. To our knowledge, no study has attempted to directly quantify the effect of fish and krill oil supplementation on warfarin TTR and bleeding incidence in a large cohort of patients taking warfarin long-term. Understanding this influence in more depth would allow for avoidable drug-interaction induced complications or alternatively the unnecessary cessation of fish and krill oil in warfarin patients by their prescriber. Therefore, the overall aim of this study was to assess the influence of fish and krill oil supplementation on warfarin control in AF and DVT patients receiving warfarin care from a private pathology practice in Queensland, Australia.

## 2. Materials and Methods

### 2.1. Study Design and Setting

A retrospective cohort analysis was conducted of patients receiving warfarin management from a large private pathology practice in Queensland. AF (any form) and DVT patients receiving long-term warfarin therapy enrolled at the private pathology clinic were eligible for study inclusion. Data collection included current medications, comorbidities, INR test results, reported bleeding and thromboembolic incidences, and patient demographics including age and gender.

### 2.2. Study Population

Data was screened for patients who had consumed fish oil or krill oil supplementation, or both, without the concurrent use of medications identified in the AMH 2015 [11] as having a known interaction with warfarin. The control group included patients who had never consumed fish or krill oil supplementation or any medications with known identified interactions with warfarin. A minimum of 30 days of INR data was required for all patients, as per testing time frames utilised in similar studies. Supplement group patients were further examined for dates of supplement use for inclusion into a ‘before, during, and after’ comparison. Exclusions for this analysis were insufficient data (i.e., less than 2 tests or less than 30 days) in each of the three categories.

### 2.3. Study Variables

Time in therapeutic range (TTR) was calculated using the Rosendaal method [4], which uses linear interpolation to assign an INR value to each day between successive observed INR values and subsequent percentage of time during which the interpolated INR values lay between 2.0 and 3.0 (or the patients’ allocated INR range). An excel template for Rosendaal TTR obtained from INR Pro Patient Anticoagulation Software was utilised for these calculations. The number of major or minor bleeding events were calculated per person-year and compared between the supplement and control group. Major bleeding was defined as bleeding that required diagnostic studies, hospitalisation, or intervention by a healthcare professional to stop bleeding (e.g., temporarily or permanently discontinuing warfarin, administration of vitamin K). Minor bleeding was defined as bleeding that did not warrant intervention or did not cause the patient to seek hospitalisation or treatment by a healthcare professional. Examples of minor bleeds included bruising, hematoma, and nosebleeds [33]. Bleeds not taken into analysis were bleeds or signs of bleeding from known causes or that could also be associated with non-warfarin patients including, but not limited to, gum bleeds, bleeding after trauma or falls, and bleeding following an operation or biopsy.

### 2.4. Statistical Methods

Subject characteristics were reported as the mean ± the standard deviation for continuous data and number and percentages for nominal or categorical data. Data was analysed using Graph Pad InStat Version 3 (Graph Pad Software, Inc., La Jolla, CA, USA), with a *p*-value of < 0.05 considered to be statistically significant. An independent Student’s *T*-Test and Mann-Whitney U test were utilised in the comparison of mean TTR between supplement and control group. The chi-squared test was utilised in the analysis of bleeding incidence per person-year.

### 2.5. Ethical Approval

Ethics approval for this project was received on 23 June 2014 by Griffith University Ethics Committee, reference number PHM/09/14/HREC.

## 3. Results

Of the 2081 patients assessed for inclusion into this study, a total of 573 warfarin users met the inclusion criteria after exclusions due to multiple interacting medications (1453 patients), less than 30 days of testing available (30 patients), and no start date of fish oil consumption (25 patients). Four hundred and twenty-eight patients were allocated to the control group, and 145 patients were allocated to the supplement group. As shown in Table 1, 416 (72.6%) patients within the total patient cohort used warfarin for AF as the primary indication, while 157 (27.4%) patients used warfarin for DVT treatment or prophylaxis. Overall, the mean age was 75.0 years (standard deviation (SD) = 14.1), with the mean age of the supplement group being 76.8 years (SD = 11.9) and 74.4 years (SD = 14.7) for the control group. Table 1 also shows that 533 (93.0%) patients were more than 50 years of age, with the supplement group having 139 (95.9%) patients aged above 50 years and the control group with 394 (92.1%) of patients aged more than 50 years. With regard to gender, overall, 316 (55.1%) patients were male, with the supplement group comprised of 51.7% males and the control group 56.3%. The three most common comorbidities in the combined patient cohort were identified to be hypertension (30.6%), diabetes (10.4%), and arthritis (9.1%).

**Figure 1:**
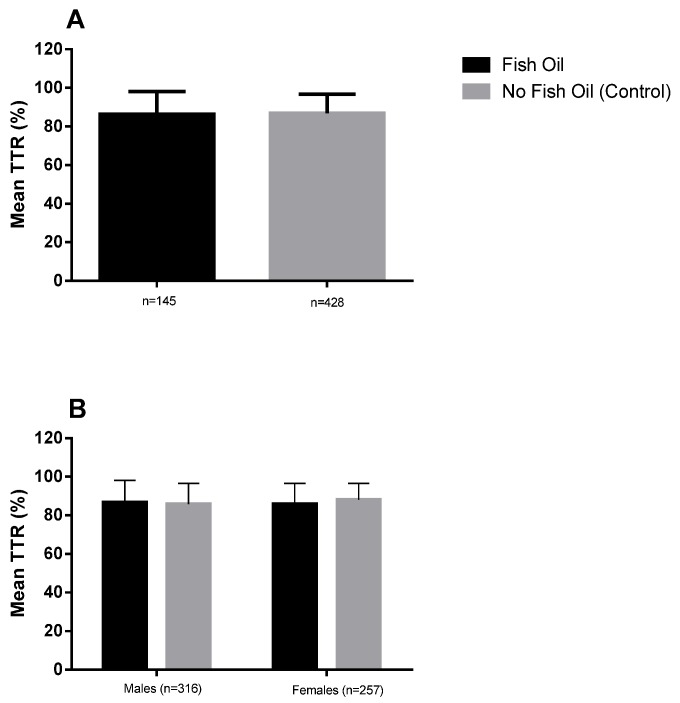
Mean TITR for (**A**) fish oil versus no fish oil supplementation; and (**B**) gender comparison for fish oil versus no fish oil supplementation. Data is shown as mean and standard deviation; males (*n* = 316), females (*n* = 257).

Individual patient data was combined to calculate mean TTR, as shown in Figure 1. Overall, no significant difference was found between the mean TTR of supplement (86.7% ± 16.0%) and control patients (86.9% ± 10.2%), with both groups exceeding the recommended target TTR of 60% by approximately 25%. Additionally, no statistically significant difference was identified between the supplement and control groups in regard to minor bleeds per person-year with an incidence of 0.079 and 0.096, respectively (Table 2). There were no major bleeds recorded for either supplement or control groups. Furthermore, no thromboembolic events occurred in either group during the studied period.

**Figure 2:**
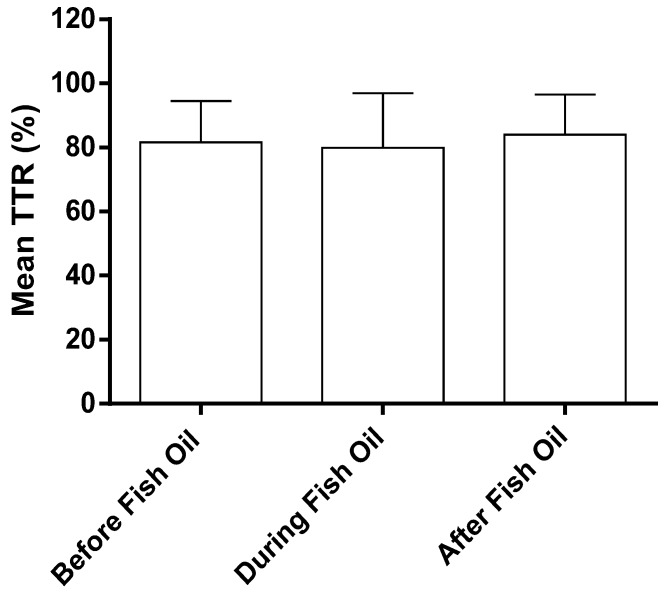
Mean TTR for patients before, during, and after fish oil supplementation. Data is shown as the mean and standard deviation, *n* = 24.

A mean TTR comparison by gender identified no differences between males and females in the supplement or control group (Figure 2). In addition, no difference was found in the mean TTR between male supplement and control patients or between female supplement and control patients. There were no statistically significant differences identified in the comparison of bleeding incidence across all groups (Table 2).

Of the 145 fish and krill oil consumers, 24 patients met the inclusion criteria for the additional analysis of mean TTR and adverse incidences of patients before, during, and after fish and krill oil consumption. These 24 patients were assessed for an average of 30 months before, 9.6 months during, and 18 months after consuming fish and krill oil. The mean TTR at various time periods was not significantly different (Figure 2). No significant differences were found in minor or major bleeding incidences in the three time periods (Table 3). Similarly, average warfarin dose did not change during this period.

## 4. Discussion

There has been conflicting evidence regarding the effect of fish and krill oil supplementation on warfarin INR, TTR, and bleeding incidence, placing many concurrent users at potential risk of dangerous therapeutic outcomes or unnecessary cessation of these supplements by their prescriber. The overall aim of this study was to assess the influence of fish and krill oil supplementation on warfarin control in AF and DVT patients. The results of this retrospective cohort study show that fish and krill oil supplementation does not significantly alter warfarin control as measured by TTR in AF and DVT patients, nor does it have an effect on the number of major or minor bleeding incidences in these patients.

This study showed no differences in mean TTR and bleeding events between fish and krill oil users and non-users in a cohort of 573 patients taking warfarin for an average of 34 months. These findings are consistent with those of other studies evaluating this interaction. Bender et al. [34] randomised a smaller group of 11 patients already consuming warfarin to receive a four-week treatment period of either placebo capsules, 3 g of fish oil daily or 6 g of fish oil daily. They reported no significant effect on the TTR of patients receiving chronic warfarin therapy and doses of 3–6 g of fish oil per day. In contrast, Jalili et al. [31] and Buckley et al. [33] both reported incidences of patients experiencing an elevation in INR, potentially resulting from the consumption of fish oil supplementation. However, these authors only reported two individual cases. Furthermore, the patient of Jalili et al. [31] had simultaneously commenced and ceased trazadone, creating difficulty in identifying the true cause of the increase in INR due to previous reports of a trazadone and warfarin interaction [35,36]. Although the patient in the case report by Buckley et al. [33] had an increase in INR following a doubling of her fish oil dose from 1000 to 2000 mg/day, followed by a fall in INR after returning to her original dose, to our knowledge, this is an isolated incident with no other similar reports to date.

In regards to the incidence of bleeding events, Bender et al. [35] also recorded the incidence of minor and major bleeding within the 11 patients they monitored over a four-week period. Similar to our study, they also reported no difference in the incidence of bleeding, with no major bleeding episodes being observed and only one bruising episode reported throughout the study. Eritsland et al. [29] also showed that concurrent use of fish and krill oil supplementation with warfarin does not have a significant effect on bleeding incidence. This study randomised 610 patients already consuming either 300 mg of aspirin daily or warfarin to receive 4 g of omega-3 fatty acids or placebo. A total of 145 warfarin patients received a placebo and a total of 174 warfarin patients received omega-3 supplementation for a period of nine months. Eritsland et al. [29] concluded that no excess in bleeding events could be accredited to the use of fish oil supplementation, even when given in addition to warfarin. The findings in our study further support the safety of omega-3 fatty acids when used in combination with warfarin therapy, in comparison to non-users. In addition to showing that there was no overall significant difference in TTR when taking fish oil supplements, we were also able to show that gender did not significantly influence TTR. To our knowledge, no other study has assessed the gender variances of fish and krill oil supplementation on warfarin control.

Omega-3 fatty acids, such as EPA and DHA found in fish and krill oil supplementation, exert their effect on the coagulation profile by two different mechanisms. Firstly, EPA and DHA replace arachadonic acid (AA), an omega-6 fatty acid, in the phospholipid membrane of platelets. As a result of less AA available for activation, a decline in thromboxane A2 occurs, causing a decrease in platelet aggregation [20,37]. A decline in the ability for platelets to aggregate increases the bleeding tendency. The second and less accepted mechanism of omega-3 fatty acids on the coagulation profile is the reduction of vitamin K-independent coagulation factor V, vitamin K-dependent factors VII, X, IX, and II (Prothrombin), and fibrinogen [38]. Evidence in some human trials have shown that these supplements can reduce the levels of the coagulation factor V and factor VII in both men and women [39,40] and of factor X in females [40]. It is thought that the reduction in the production of these clotting factors may arise from the lowering of lipoproteins, which are necessary for the transport of vitamins K1 and K2 in plasma [41]. As coagulation factors II, VII, IX, and X require γ-carboxylation by vitamin K for biological activity, a decline in the availability of vitamins K1 and K2 would result in a hypo-coagulant state [14]. The prothrombin time (PT) utilised in the calculation of patient INR measures the activity of extrinsic (tissue factor) and common pathways of coagulation, and is therefore dependent on the functional activity of clotting factors VII, X, V, II (Prothrombin) and fibrinogen. A decline in the concentration of factors VII, V, and X (in females) and fibrinogen by omega-3 fatty acids may result in an increase in PT, causing an increase patient INR and potential decrease in TTR.

Although there have been in vitro studies that have supported the effect of fish and krill oil supplementation on prolonged bleeding and coagulation factors, the majority of human studies do not support this effect. Previously, there had been conflicting evidence as to whether fish and krill oil increased INR and bleeding incidence when used in conjunction with warfarin. Our study suggests that there is no significant effect on warfarin TTR and bleeding caused by fish and krill oil supplementation, and that it is therefore safe for warfarin users to concurrently consume warfarin and fish and krill oil supplements. However, when taking into consideration the results obtained in our study, it should be noted that all of our patients were managed by a warfarin clinic. Bernaitis et al. [42] reported the Queensland warfarin care program can achieve high TTRs regardless of the influence of numerous factors including gender, age, and socioeconomic status. Chiquette et al. [43] further showed patients managed in a private clinic have a higher baseline level of warfarin control than those managed community practice. Due to this, patients not managed by a private warfarin clinic may experience more significant changes in warfarin control and adverse events than shown in our study. Future directions for this study should involve the analysis of community-managed warfarin patients to ascertain the influence of fish and krill oil supplementation on warfarin control and bleeding incidence in patients with potentially poorer overall TTR. Furthermore, the absence of individual fish and krill oil doses did not allow for the analysis of potential dose-effect on warfarin to be conducted in our study, so we were unable to establish whether varying doses of fish and krill oil affect warfarin TTR and bleeding incidence. Assessing the influence of various concentrations of EPA and DHA on TTR would further assist in identifying potential dose related influence on INR and TTR. In conclusion, the results of this study show that omega-3 supplementation, such as fish oil and krill oil, do not significantly influence warfarin control or bleeding incidence in AF and DVT patients managed at a private pathology practice in comparison to non-fish and -krill oil users. 

## 5. Conclusions

Warfarin is a widely prescribed anticoagulant requiring monitoring with TTR linked to both therapeutic efficacy and adverse effects. Numerous factors are known to influence TTR including drug interactions. However, there is limited information on interactions with complementary and alternative therapies including the widely used fish and krill oils. Fish and krill oil supplementation had no significant influence on both warfarin TTR control, bleeding incidence, or thromboembolic events in AF and DVT patients achieving well controlled warfarin therapy through management at an anticoagulant clinic.

## Figures and Tables

**Table 1:** Demographic characteristics of patient cohort.

Variable—*n* (%)	All Patients	Fish Oil	No Fish Oil
	(*n* = 573)	(*n* = 145)	(*n* = 428)
**Age (years)**			
**Mean (Standard Deviation)**	75.0 (14.1)	76.8 (11.9)	74.4 (14.7)
≤25	2 (0.3)	0	2 (0.5)
26 to ≤50	38 (6.6)	6 (4.1)	32 (7.5)
51 to ≤75	198 (34.6)	50 (34.5)	148 (34.6)
75 to ≤100	335 (585)	89 (61.4)	246 (57.5)
**Gender**			
Male	316 (55.1)	75 (51.7)	241 (56.3)
Female	257 (44.9)	70 (48.3)	187 (43.7)
**Comorbidities**			
AF	416 (72.6)	113 (77.9)	303 (70.8)
DVT	157 (27.4)	32 (22.1)	125 (29.2)
HTN	165 (30.6)	37 (29.4)	128 (31.0)
Diabetes	56 (10.4)	13 (10.3)	43 (10.4)
Hypercholesterolemia	28 (5.2)	6 (4.8)	22 (5.3)
Angina	9 (1.5)	3 (2.4)	5 (1.2)
HF	8 (1.5)	-	8 (1.9)
IHD	29 (5.4)	9 (7.1)	20 (4.7)
COPD	16 (3.0)	1 (0.8)	15 (3.6)
Asthma	16 (3.0)	4 (3.2)	12 (2.9)
Dyslipidaemia	20 (3.7)	4 (3.2)	16 (3.9)
Gout	18 (3.3)	3 (2.4)	15 (3.6)
Cancer	28 (3.3)	4 (3.2)	14 (3.4)
GORD	27 (5.0)	8 (6.3)	19 (4.6)
Arthritis	49 (9.1)	13 (10.3)	36 (8.7)
Renal Failure	9 (1.7)	3 (2.4)	6 (1.5)
OP	23 (4.3)	4 (3.2)	19 (4.6)

AF = Atrial Fibrillation; DVT = Deep Vein Thrombosis; HTN = Hypertension; HF = Heart Failure; IHD = Ischaemic Heart Disease; COPD = Chronic Obstructive Pulmonary Disease; GORD = Gastric Oesophageal Reflux Disease; OP = Osteoporosis.

**Table 2:** Bleeding incidence per person-year for (A) supplement and control group patients, and (B) gender comparisons for supplement and control groups.

Bleeding Event	Supplement Group		Control Group	
**A**				
Minor	0.07		0.09	
Major	0		0	
**B**				
	Males	Females	Males	Females
Minor	0.04	0.05	0.10	0.08
Major	0.00	0.01	0.01	0.00

**Table 3:** Bleeding incidence per person-year for patients before, during, and after supplementation.

Bleeding Event	Before Fish Oil	During Fish Oil	After Fish Oil
Minor	0.16	0.16	0.22
Major	0.01	0.00	0.00
